# Deep Learning Algorithms Achieved Satisfactory Predictions When Trained on a Novel Collection of Anticoronavirus Molecules

**DOI:** 10.3389/fgene.2021.744170

**Published:** 2021-11-29

**Authors:** Emna Harigua-Souiai, Mohamed Mahmoud Heinhane, Yosser Zina Abdelkrim, Oussama Souiai, Ines Abdeljaoued-Tej, Ikram Guizani

**Affiliations:** ^1^ Laboratory of Molecular Epidemiology and Experimental Pathology-LR16IPT04, Institut Pasteur de Tunis, Université de Tunis El Manar, Tunis, Tunisia; ^2^ Laboratory of BioInformatics BioMathematics and BioStatistics (BIMS)-LR20IPT09, Institut Pasteur de Tunis, University of Tunis El Manar, Tunis, Tunisia; ^3^ Engineering School of Statistics and Information Analysis, University of Carthage, Ariana, Tunisia

**Keywords:** deep learning, artificial neural network, SARS-CoV-2, machine learning, graph convoluational networks, drug discovery and repurposing

## Abstract

Drug discovery and repurposing against COVID-19 is a highly relevant topic with huge efforts dedicated to delivering novel therapeutics targeting SARS-CoV-2. In this context, computer-aided drug discovery is of interest in orienting the early high throughput screenings and in optimizing the hit identification rate. We herein propose a pipeline for Ligand-Based Drug Discovery (LBDD) against SARS-CoV-2. Through an extensive search of the literature and multiple steps of filtering, we integrated information on 2,610 molecules having a validated effect against SARS-CoV and/or SARS-CoV-2. The chemical structures of these molecules were encoded through multiple systems to be readily useful as input to conventional machine learning (ML) algorithms or deep learning (DL) architectures. We assessed the performances of seven ML algorithms and four DL algorithms in achieving molecule classification into two classes: active and inactive. The Random Forests (RF), Graph Convolutional Network (GCN), and Directed Acyclic Graph (DAG) models achieved the best performances. These models were further optimized through hyperparameter tuning and achieved ROC-AUC scores through cross-validation of 85, 83, and 79% for RF, GCN, and DAG models, respectively. An external validation step on the FDA-approved drugs collection revealed a superior potential of DL algorithms to achieve drug repurposing against SARS-CoV-2 based on the dataset herein presented. Namely, GCN and DAG achieved more than 50% of the true positive rate assessed on the confirmed hits of a PubChem bioassay.

## 1 Introduction

Discovery and design of effective treatments against COVID-19 is actually an active research field. Tremendous efforts have been deployed worldwide to find new molecules with therapeutic potential against its pathogenic agent SARS-CoV-2 (Song et al., 2021). The most forerunner achievements mainly consisted in drug repurposing attempts of previously described drugs able to affect the SARS-CoV such as chloroquine and its derivatives (Vincent et al., 2005; Pastick et al., 2020; Yao et al., 2020; Galan et al., 2021; Moiseev et al., 2021). Other antivirals or antibiotics were also assessed for their potential as COVID-19 therapeutics (Pillaiyar et al., 2020; Kelleni, 2021). Still, as of today, no candidates have been yet retained as a universal COVID-19 treatment (Hoffmann et al., 2020; Dragojevic Simic et al., 2021). Various approaches were adopted, including computational methods toward a faster discovery of drugs, given the urge of the global sanitary situation.

Computational approaches may be split into two subcategories: Structure-Based Drug Discovery (SBDD) and Ligand-Based Drug Discovery (LBDD). For SBDD, the structure of a molecular target is used to perform virtual screenings of large chemical libraries. The most popular targets are the Spike protein, known as the S protein, the 3-Chymotrypsin-Like cysteine protease (3CLpro), also called the main protease (Mpro), and the Papain-Like protease (PLpro) (Chellapandi and Saranya, 2020; Trezza et al., 2020; Zhang et al., 2020; Zhai et al., 2021). These approaches rely on the availability of structural data of SARS-CoV-2 proteins, which are noticeably abundant as compared to other organisms. In fact, as of July 14, 2021, the RCSB PDB database accounted for 446 structures of the S protein and its binding domains, 360 crystal structures of the 3CLpro, 35 for the PLpro, and 505 structures corresponding to other SARS-CoV-2 proteins (RCSB).

On the other hand, LBDD is more likely dependent on the availability of data on the biological activity of molecules. Machine learning (ML) approaches demonstrated their ability to predict the activity of novel molecules based on such data (Altae-Tran et al., 2017; Lo et al., 2018; Vamathevan et al., 2019; Zeng et al., 2019; Korkmaz, 2020). The underlying assumption is that chemically and topologically similar compounds may have similar bioactivities and targets (Gfeller et al., 2014; Shi et al., 2015; Perualila-Tan et al., 2016). These approaches were extensively used in novel drug discovery (DD) and repurposing against COVID-19 (Keshavarzi Arshadi et al., 2020; Bung et al., 2021; Yang et al., 2021). In fact, dedicated resources have been developed to facilitate and enhance international efforts toward DD against COVID-19. Namely, the COVID-19 Moonshot Consortium has deployed international efforts in tackling data collection and curation of molecules targeting the 3CLpro of SARS-CoV-2. Their approach allied with SBDD and LBDD techniques (Achdout et al., 2020). In fact, data availability is a cornerstone in building reliable ML models. This being said, data in DD is often sparse, heterogeneous, noisy, or too few. Multiple efforts have been made to build ML algorithms able to deal with such limitations and achieve satisfactory predictions (Duran-Frigola et al., 2019; Irwin et al., 2020; Yang et al., 2020).

Beyond COVID-19 research, ML and deep learning (DL) were applied to a variety of DD projects. Applications can be split into two types: 1) activity prediction through regression and 2) classification of molecules into classes, mostly active vs. inactive (Rifaioglu et al., 2019; Vamathevan et al., 2019). ML algorithms are implemented and trained on binary or float values descriptors of a fixed length, generated using a chemical structure encoding system. The most popular encoding systems are either the physicochemical descriptors (molecular weight, H-bond donors, H-bond acceptors, rotatable bonds, etc.) or molecular fingerprints (Jing et al., 2018). The latter correspond to a variety of algorithms that are able to capture topological features and properties within chemical structures. Most of them calculate a series of binary digits that encode the presence or the absence of particular substructures in the molecule. More recently, there was a rising interest in graph convolution networks as chemical structure encoding systems in the frame of DL applications in LBDD (Micheli, 2009; Lusci et al., 2013; Duvenaud et al., 2015; Kearnes et al., 2016; Altae-Tran et al., 2017). A molecular graph is the most common machine-readable representation (David et al., 2020). Chemical representations in these schemes lie in mapping the atoms and bonds of a molecule into sets of nodes and edges. Spatial relationships between the nodes are then encoded through network embedding. This leads to a low-dimensional vector representation of the molecular graph, preserving both network topology structure and node content information (Wu et al., 2020). Graph convolutional networks (GCN) apply then a series of convolution layers to construct the whole molecule encoder. Graphs have irregular designs and sizes; there is no spatial order attached to the nodes. As a result, traditional convolution on regular grid-like structures cannot be applied directly to graphs. In the literature, efforts have been made to generalize the convolution operator to non-Euclidean structured data, resulting in convolutional graph networks (CGNs). GCNs have emerged as the state-of-the-art encoding when it comes to DD (Sun et al., 2020), especially when one seeks to extract features with respect to the data structure. This extraction is done automatically from raw inputs (Lavecchia, 2019). Duvenaud et al. presented a graph convolution method to encode molecule structures using a differentiable neural network (NN) that generalizes fingerprint-based features *via* backpropagation on an undirected graph representation of the molecule (Duvenaud et al., 2015). The authors demonstrated that applying graph convolution enhances property predictions as compared to conventional circular fingerprints. Kearnes et al. also described a graph convolution approach that learns from a graph representation of the molecule while taking into account its structure and composition (Kearnes et al., 2016).

Here, we present a dataset of molecules validated for their effects on SARS-CoV-2 and/or SARS-CoV through viral growth inhibition, cell-based, or enzymatic experiments. Data were collected through an extensive search of the literature and databases, curated and formatted for cheminformatics simulations toward LBDD against COVID-19. Chemical structures of the molecules were then encoded through multiple systems to be readily useful as input to conventional ML algorithms or for GCN. We run an extensive set of simulations under different splitting and formatting conditions of the data to identify the ML and DL algorithms that could achieve satisfactory results. Most promising models were then optimized, and their performances were validated through cross-validation. An external validation step was performed to assess the potential of these algorithms to achieve drug repurposing using experimental data on the FDA-approved drugs collection.

## 2 Materials and Methods

### 2.1 Data Collection

The data collection process included three distinct approaches. The first consisted in literature mining. We collected data on molecules described in peer-reviewed papers as anticoronavirus effectors. Two beta-coronavirus species were considered: SARS-CoV (Severe Acute Respiratory Syndrome Coronavirus, 2003) and SARS-Cov-2 (Severe Acute Respiratory Syndrome Coronavirus, 2019). The second approach consisted in retrieving data on molecules deposited in the RCSB PDB as cocrystals with SARS-CoV and SARS-CoV-2 proteins, mainly the 3CL-protease and the Papain-Like protease. When available, activity data on these cocrystallized inhibitors were fetched from corresponding scientific publications. The third approach consisted in retrieving data from bioassays deposited in the PubChem database (Kim et al., 2020). Priority was given to bioassays targeting SARS-CoV-2 or related molecular targets, with a special interest in large bioassays on other coronaviruses. Data collected from these bioassays correspond to viral growth inhibition or cell-based tests targeting a given viral enzyme. In total, data from 10 COVID-19 bioassays were included. These were complemented by four bioassays targeting SARS-CoV. PubChem IDs of the bioassays, their types, and sizes are listed in Supplementary Table S1. Bioassay datasets were then formatted to be merged with the literature dataset previously collected. Data collected on each molecule included chemical structure, name, chemical name if indicated, activity, target virus, and any additional information such as identifiers in the PubChem database, *in vitro* IC_50_ values, cellulo IC_50_ values, and any other valuable biological data (*in vivo* EC_50_, inhibition rate at a given concentration, etc.). The chemical structure of the molecules was encoded using the Simplified Molecular Input Line Entry System (SMILES). For compounds with a graphical description of their structure in the literature, we used the Optical Structure Recognition Application (OSRA) tool (Filippov and Nicklaus, 2009) to correctly infer the corresponding SMILES. For compounds referred to in the literature using a common name, SMILES were directly retrieved from the PubChem database. Duplicates were removed using a similarity threshold of 97% based on the Tanimoto coefficient. Each molecule was assigned an activity status that can be “active,” “inactive,” or “inconclusive.” For molecules retrieved from PubChem bioassays, these status values were provided from the experimentalists’ data. For molecules fetched in the literature, these status values were deduced from the authors’ conclusions. For the molecules retrieved from the PDB records, these status values were assigned to “active” by default. In fact, we considered that the ability of a molecule to bind to a given protein receptor encloses valuable information on potential active moieties, although no biological activity is reported for these molecules. Any data point with inconclusive or blurry value was discarded for robustness sake.

### 2.2 Datasets Construction

The benchmark datasets used herein were split using two different approaches. First, a random split with no consideration for chemical equilibration among the training, validation, and test sets was applied. Then, a scaffold split (Ramsundar et al., 2019) was applied. The scaffold split method would cluster molecules based on the Murcko scaffold calculated using RDkit. Compounds with different scaffolds are placed into different sets (Ramsundar et al., 2019). This significantly reduces the overlap of chemical scaffolds between the training and the test sets (Ramsundar et al., 2019).

In addition, we tested how the size of the validation and test sets would affect the algorithms’ performances. Thus, we tested two scenarios: 80/10/10 and 60/20/20 split. An additional splitting method of the original dataset that permitted the generation of category-specific subsets for validation purposes was applied. Undersampling and oversampling were applied in order to obtain equilibrated datasets in each case. Undersampling consisted in reducing the inactive molecules subset to achieve equilibrated classes. Oversampling consisted in artificially generating additional SMILES of the active molecules in order to reach the inactive subset size.

### 2.3 Molecular Structure Embedding

Based on the SMILES, we calculated either molecular fingerprints or graph convolution-based features that consist in binary or float values vectors to be used as input to the ML and DL algorithms, respectively. As fingerprints, we chose the extended-connectivity fingerprints with a radius of two atoms (ECFP4), also known as the circular Morgan fingerprints (Rogers and Hahn, 2010), to encode the molecule structures for ML algorithms. We used the RDkit library to generate 2,048-bit length ECFP4. Molecules with erroneous SMILES or chemistry were removed at this stage. We used these fingerprints to calculate the Tanimoto coefficient of similarity in a pairwise fashion. This metric consists of the fraction of the intersection over the union of the set of chemical substructures between two molecules. It is one of the most used to assess the chemical similarity between molecules (Chung et al., 2019). As for the graph convolution-based features, depending on the DL architecture requirement, two featurizers were used:

• The ConvMolFeat featurizer (Duvenaud et al., 2015) to generate input for the Graph Convolutional (GraphConv) (Duvenaud et al., 2015) and the Directed Acyclic Graph (DAG) (Lusci et al., 2013) models.
• The MolGraphConvFeat (Kearnes et al., 2016) to generate input for the GAT (Velickovic et al., 2018) and the GCN (Kipf and Welling, 2016) models.

Graphical convolutional models map molecules as undirected graphs whose vertices and edges represent individual atoms and bonds, respectively. Graphical convolutions extract meaningful patterns from basic descriptions of graph structure (atom and bond properties and graph distances) to form molecule-level representations. They are considered fully integrated approaches to virtual screening. The output of the model is invariant to the order in which the atom and bond information is encoded in the input. The graph represents class similarity information and is fed into DL classification models.

### 2.4 ML and DL Algorithms

We implemented multiple artificial intelligence (AI) algorithms to develop classification models: ML, ensemble learning methods (EL), and DL. We implemented seven ML algorithms, out of which two are simple ML algorithms, namely, Logistic Regression (LR) and Support Vector Machine (SVM). Five additional EL algorithms were implemented, namely, Random Forests (RF), Multitask Classifier (MTC), IRV-MTC, Robust MTC, and Gradient Boosting (XGBoost). EL are learning algorithms that construct the first set of classifiers and then construct a new one by taking a weighted vote of data predictions from the previous classifiers (Dietterich, 2000). These algorithms were implemented under Scikit-learn, an open-source python library (Pedregosa et al., 2011). LR measures the relationship between a categorical dependent variable and one or more explanatory variables. This is performed by estimating probabilities using a logistic function, which is the cumulative logistic distribution, thus predicting the probability of certain outcomes. The SVM is one of the most popular supervised ML algorithms. It is effective in high-dimensional spaces. The hyperplane learning in the SVM algorithm can be performed using different kernel functions for the decision function. The RF method is an ensemble method, based on decision trees. The model fits on various subsamples of the dataset and uses averaging to improve predictive accuracy and control overfitting. The Gradient Boosting model implemented herein is called XGBoost (Paul et al., 2020). It is an extremely gradient boosting algorithm and a decision tree-based boosting integration algorithm (Ericksen et al., 2017). Further ensemble methods have been tested: Multitask Classifier (MTC), IRV-MTC, and Robust MTC. These are fully connected NN, where various hyperparameters are optimized. They operate like EL algorithms, where they integrate data from different tasks to achieve classification. When used on a single task data, they are a nonlinear classifier that performs repeated linear and nonlinear transformations on one single task (Ramsundar et al., 2017).

Then, four DL architectures were implemented under the DeepChem library (Ramsundar et al., 2019): the Graph Convolutional Model (GraphConv), the DAG model, the Graph Attention Networks model (GAT), and the GCN model. The GraphConv Model (Duvenaud et al., 2015) learns a vector representing the compound from the graph-based representation of the molecule. It predicts the target value directly through graph convolution operations. Convolutional networks operate the same operation locally and globally and combine the information in a common pooling step. Feature extraction involves computing an initial feature vector and a list of neighbors for each atom. The feature vector summarizes the local chemical environment of the atom, including atomic types, hybridization types, and valence structures. The neighbor lists map the connectivity of the entire molecule and are then processed in each model to generate graph structures (Wu et al., 2018). The DAG model is an ensemble of recursive NN that associate all vertex-centered acyclic orientations of the graph representation of the molecule. It is slightly dependent on the molecular descriptors since suitable representations are learned from the DAG representation (Lusci et al., 2013). The graph attentional layer (GAT) model (Velickovic et al., 2018) is a convolutional NN that operates on graph-structured data, taking advantage of self-attention hidden layers. The attention mechanism is applied in a shared manner to all edges of the graph and thus does not depend on prior access to the overall structure of the graph or to (characteristics of) all its nodes. It allows assigning (implicitly) different importance to the nodes of the same neighborhood. GCN is an implementation of graph convolutional NN (Kipf and Welling, 2016). It learns hidden layer representations that are able to encode both individual features of nodes and their respective environments. It computes a weighted sum of the node representations in the graph, where the weights are computed by applying a gating function to the node representations, and then applies a max pooling of the node representations. It perform the final prediction using a multilayer perceptron (MLP) over a concatenation of the last convolution layer output. It differs from the GraphConv model by the fact that, for each graph convolution, the learnable weight in this model is shared across all nodes. The GraphConv model computes separate learnable weights for nodes.

Under the DeepChem library, both the GraphConv Model and the DAG model were implemented to learn from MolConv featurizer (Duvenaud et al., 2015) that corresponds to GCN that learns from circular morgan fingerprints-like representation of the molecule. On the other hand, the GAT and GCN models have been implemented in a way that they can learn from the MolGraphConv featurizer (Kearnes et al., 2016). Data were split into training, validation, and test sets. The hyperparameters of the DL models were tuned using the loss of the validation sets.

### 2.5 Model Evaluation and Selection

We performed the first comparison of all models’ performances with hyperparameters set to the optimal values obtained through the MoleculeNet benchmarks (Wu et al., 2018). To better evaluate the different models, we calculated multiple performance metrics, including the ROC-AUC, accuracy, F1-score, Matthews correlation coefficient (MCC) (Matthews, 1975), and Cohen’s Kappa coefficient (*κ*). Then, we performed a cross evaluation of the model performances when trained and tested on stratified subsets of the data based on the different categories of targets. Accuracy, F1-score, Recall, and specificity were used as evaluation metrics for these simulations.

For the metric definitions, the following abbreviations are used: the number of true positives (TP), the number of false positives (FP), the number of true negatives (TN), and the number of false negatives (FN). Specificity, also called the False Positive Rate (FPR), is the model’s ability to correctly reject an inactive molecule. Specificity of a test is the proportion of molecules that are truly inactive, which are classified as is. It is defined as follows:

$$Speci\mathrm{fi}city=\frac{TN}{TN+FP}.$$ (1)

Model Recall can be thought of as the percentage of true class labels correctly identified by the model as true. It is equal to the model sensitivity in binary classification and is also called the True Positive Rate (TPR). It is defined as follows:

$$Recall=\frac{TP}{TP+FN}.$$ (2)

The F1-score is the harmonic mean of the Recall and precision:

$$\text{F}1-\text{score}=2*\frac{Recall*Precision}{Recall+Precision}.$$ (3)

where precision is the probability of a predicted true label is predicted as true and is defined as follows:

$$Precision=\frac{TP}{TP+FP}.$$ (4)

Accuracy is the percentage of correctly identified labels out of the entire population.

$$Accuracy=\frac{TP+TN}{TP+FP+TN+FN}.$$ (5)

The ROC-AUC score tells how much the model is capable of distinguishing between classes. It varies between 0 and 1, where 1 means a perfect prediction. The MMC is a correlation coefficient between the observed and predicted binary classifications. It is between −1 and +1, where +1 indicates a perfect prediction, 0 indicates no better than random, and −1 indicates prediction and observation are totally different.

$$MMC=\frac{\left(TP*TN\right)-\left(FP*FN\right)}{\sqrt{\left(TP+FP\right)*\left(TP+FN\right)*\left(TN+FP\right)*\left(TN+FN\right)}}.$$ (6)

Cohen’s Kappa method measures interclassifier agreement in qualitative classification tasks. It evaluates the agreement between two classifiers and takes into account the random occurrence of the agreement. A value close to one denotes better agreement between the results and ground truth.

$$\kappa =\frac{2*\left(TP*TN-FN*FP\right)}{\left(TP+FP\right)*\left(FP+TN\right)+\left(TP+FN\right)*\left(FN+TN\right)}.$$ (7)

The best performers were then selected for hyperparameter optimization on the particular anticoronavirus dataset collected through the present study. Their performances were mainly assessed through ROC-AUC, F1-score, Recall, Accuracy, MCC, and Cohen’s Kappa scores, which are a set of popular metrics in evaluating ML algorithms in a variety of applications (Le et al., 2019; Le and Huynh, 2019; Le and Nguyen, 2019). ML algorithm optimization included all optimizable parameters for the respective model. For DL architectures, the number of epochs, the batch size, the learning rate, the dropout, or the number of graph features when they apply were optimized. We selected the configuration that maximizes the ROC-AUC of the model on the validation set. The accuracy, the F1-score, the MCC, and Cohen’s Kappa coefficient were also calculated for all combinations.

Tenfold cross-validation was performed, and the mean ROC-AUC, F1-score, and Recall values were reported. A stratified validation was also applied in order to assess the ability of the algorithms trained on the heterogeneous dataset to correctly predict active molecules from different categories of experiments. The sensitivity (Recall) and specificity were herein used as performance indicators. The optimized models were then subject to an external validation using an unseen set of molecules. We used a PubChem bioassay that consisted in a primary screen of 1,518 FDA-approved molecules against SARS-CoV-2-infected cells (AID_1409594). A total number of 17 hits were retained as potentially active molecules, and their antiviral efficacy was further confirmed through a second assay (AID_1409595). We performed a prediction of these 1,518 FDA-approved drugs as anti-SARS-CoV-2 inhibitors using the best performing algorithms.

## 3 Results

### 3.1 Integration Efforts Led to a Curated Dataset of Anticoronavirus Molecules

**FIGURE 1:**
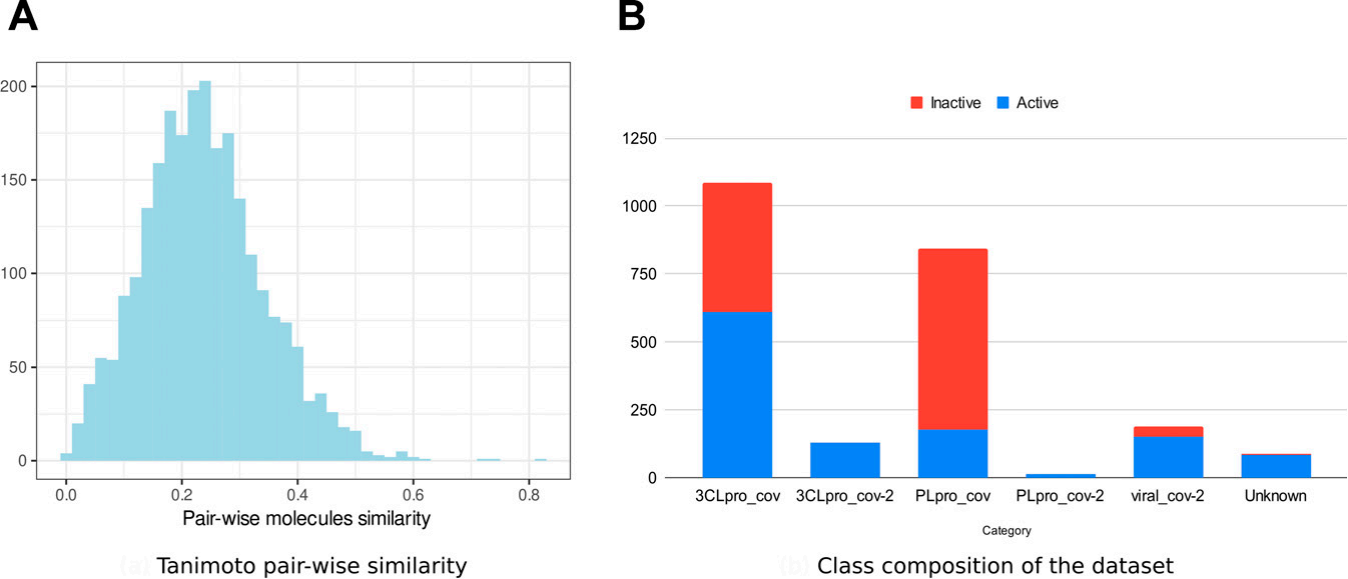
Anticoronavirus dataset composition. **(A)** Distribution of the pairwise chemical similarity among the molecules based on the Tanimoto coefficient. **(B)** Proportions of “active” and “inactive” molecules within each experimental category.

We collected data on molecules with anticoronavirus effects, out of which 533 were retrieved from literature. All remaining compounds were collected from 14 PubChem bioassays. Since activity types were different from one source to the other, we considered the activity as a binary variable. Initially, four classes of activity status were listed: active, inactive, unspecified, and inconclusive. Only molecules within the first two classes were retained in the frame of the present work. The combined set of active and inactive molecules was subject to redundancy check, and duplicates were removed. The number of active molecules was equal to 1,305 at this stage. We then looked to obtain an equal number (1,305) of inactive molecules, which were in larger numbers, namely, within large bioassays. Thus, from some SARS-CoV bioassays, only a subset of inactive molecules was randomly selected (see Supplementary Table S1). Ultimately, 2,610 nonredundant compounds were obtained. We performed a structural similarity analysis to assess the chemical diversity of the dataset (Figure 1). Based on the circular Morgan fingerprints, we calculated the pairwise distance between all compounds using the Tanimoto similarity coefficient. The similarity distribution demonstrated too few values higher than 60%. This indicates a high chemical diversity within the dataset. Also, experiments that revealed these molecules included enzymatic activity assays against one of the viruses proteases 3CLpro and PLpro, inhibition assays targeting the whole virus, and cell-based assays. We defined most relevant experiment categories as follows: 3CLpro_cov, 3CLpro_cov2, PLpro-cov, PLpro_cov2, and viral_cov2. Each category presents a specific count in terms of active and inactive molecules (Figure 1), revealing unbalanced and insufficient data within some categories. Within the molecules with known molecular targets, only 0.7% were targeting the PLpro of SARS-CoV-2, while 40.6% were targeting PLpro of SARS-CoV (Figure 1). The remaining molecules were targeting the 3CLpro of SARS-CoV-2 (6.3%) and 3CLpro SARS-CoV (52.4%). This bias reflects the higher interest toward the 3CLpro as a therapeutic target against coronaviruses (Yang et al., 2021; Zhai et al., 2021).

### 3.2 Graph Convolution-Based Models Compete With Baseline ML Algorithms

At this stage, we disposed of 2,610 anticoronavirus molecules. We used a random and a scaffold split of the dataset using two splitting proportions of the training, validation, and test sets as follows: 80/10/10 and 60/20/20. We seek to identify which scenario is overall optimal. The final datasets, ready for the upcoming experiments, are available on GitHub.

We first run preliminary simulations of seven ML algorithms and four DL algorithms using the hyperparameter values released by the MoleculeNet authors (Wu et al., 2018). These optimized values were tuned on multiple types of datasets related to DD tasks. Test set representing 10% of the dataset derived significantly better results than test sets of size 20% (Supplementary Table S2). This highlighted the need to keep the training set at its higher size in order to reach satisfying levels of training. Such proportions also demonstrated the highest scores of few-shot learning algorithms (Liu et al., 2021).

**FIGURE 2:**
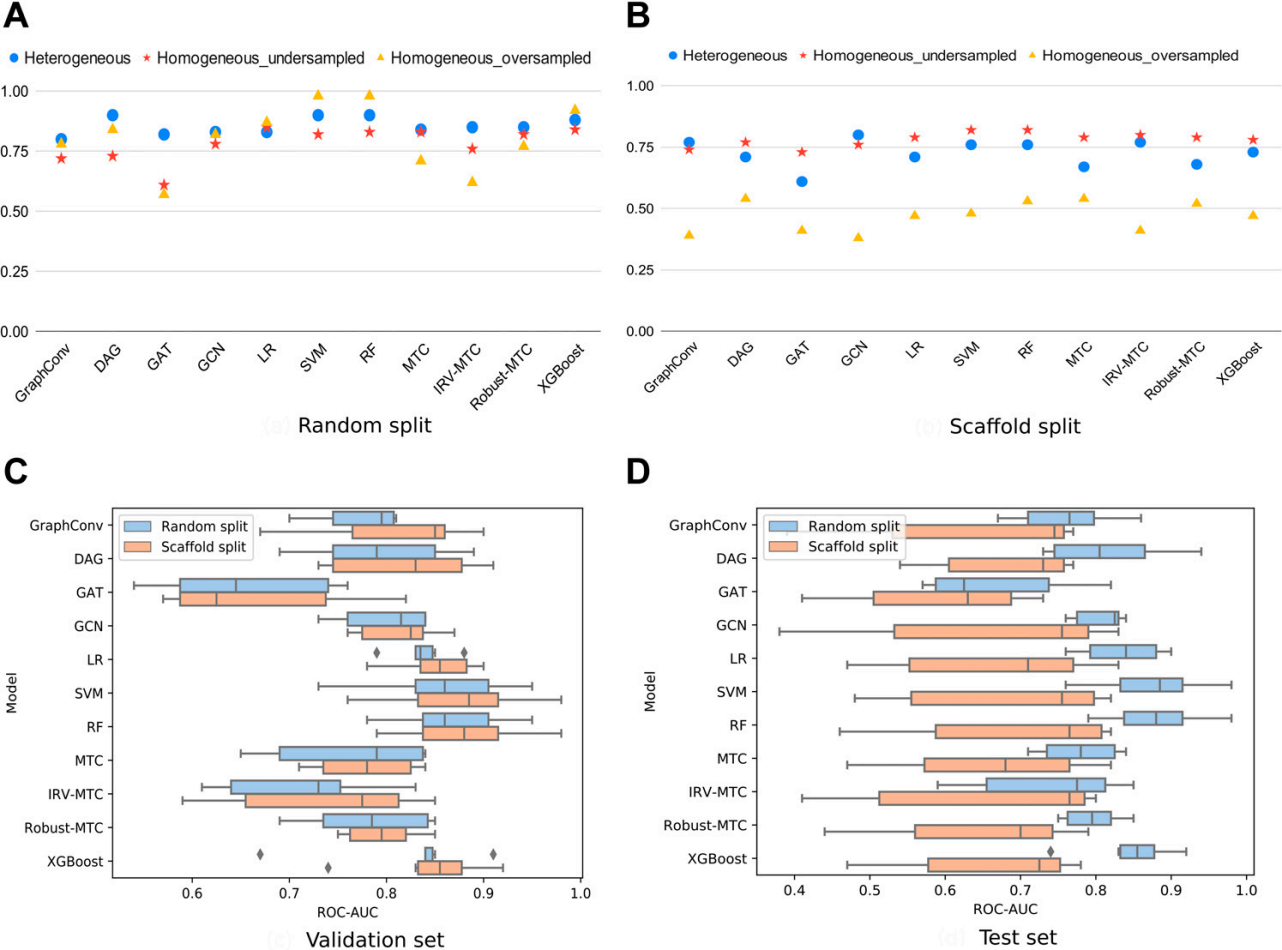
ROC-AUC scores of all models for three different datasets (heterogeneous, undersampled homogeneous, and oversampled homogeneous). **(A)** ROC-AUC scores achieved by all models under the random 80/10/10 split. **(B)** ROC-AUC scores achieved by all models under the scaffold 80/10/10 split. **(C)** Boxplots of the ROC-AUC scores achieved by each model on all validation subsets (heterogeneous, undersampled homogeneous, and oversampled homogeneous included) and with both splitting proportions (80/10/10; 60/20/20). **(D)** Boxplots of the ROC-AUC scores achieved by each model on all test subsets (heterogeneous, undersampled homogeneous, and oversampled homogeneous included) and with both splitting proportions (80/10/10; 60/20/20).

To better understand to which extent the heterogeneity of our dataset may be influential, we considered a subset of homogeneous data from the largest PubChem bioassay on SARS-CoV within our dataset: AID_1706. It is a biochemical assay targeting the enzymatic activity of the 3CLpro of SARS-CoV, through which 290,893 compounds were tested. A total of 405 molecules showed an inhibitory effect on the 3CLpro-mediated peptide cleavage. Based on this bioassay, we generated one undersampled (810 molecules) and one oversampled (2,430 molecules) homogeneous datasets. On randomly split data, ROC-AUC scores on the heterogeneous dataset were the most stable across the different algorithms. The best results were exhibited by RF and SVM on the oversampled homogeneous dataset. For the DL algorithms, GraphConv model, DAG, and GCN demonstrated satisfying performances ( > 80%) on the oversampled and heterogeneous datasets, with comparable values. Overall, six out of eleven presented similar ROC-AUC scores between the heterogeneous and the oversampled homogeneous datasets (Figure 2A). Noticeably, these datasets had comparable sizes and were larger than the undersampled homogeneous dataset. This confirms the sensitivity of the AI models’ performances to the dataset’s size (Yang et al., 2020).

On scaffold-based split datasets, ROC-AUC scores were lower than those obtained with the randomly split data (Figure 2B). Moreover, the lowest values were observed for the oversampled homogeneous data, while the highest were obtained with the undersampled homogeneous data. The heterogeneous dataset achieved scores comparable to the undersampled dataset varying between 61 and 80%. This scheme was observed in overall simulations (Figures 2C,D). The difference between scores obtained with the oversampled and the heterogeneous datasets, at equal sizes, indicated a lower chemical diversity (number of scaffolds) within the homogeneous dataset. Thus, scaffold splitting induced lower diversity across the train and the test sets, which points out the interest of using a random split of the heterogeneous dataset in building performing ML/DL models. For the upcoming simulations, we will report results on the heterogeneous dataset using an 80/10/10 random split.

The scores of the training, the validation, and the test sets obtained with all splitting combinations showed little to no overfitting, as no significant differences were observed between these sets’ scores overall (Table 1). According to the ROC-AUC scores on the test set, RF and SVM were the best classifiers within the ML/EL algorithms (Figure 2). Although the Multitask Classifier (MTC) and its variants IRV and Robust MTC exhibited higher Recall, they exhibited lower values of ROC-AUC and F1-score. We concluded that RF and SVM were the most likely to correctly predict the active molecules as being active. In the set of DL architectures, the DAG and the GCN models were the best performers. They both achieved ROC-AUC scores of 87%, F1-scores of 73 and 79%, and Recall values equal to 68 and 82%, respectively (Table 1). Noticeably, the DAG model had quite higher performances on the train set (99% for all metrics). This was not the case for the GCN. This indicated that the herein used hyperparameters for the DAG model were close to the optimal configuration for our case study. We may expect better results for the GCN algorithm after the optimization step. For the upcoming steps, we will consider the RF, the DAG, and the GCN models for hyperparameters tuning and optimization.

**TABLE 1:** Performances of 11 algorithms in predicting activity class of the anticoronavirus dataset. Optimized settings based on the MoleculeNet benchmarks were considered for all models.

Model	Train	Validation	Test	Train	Validation	Test	Train	Validation	Test
	ROC-AUC	ROC-AUC	ROC-AUC	F1-score	F1-score	F1-score	Recall	Recall	Recall
GraphConv	0.99	0.80	0.86	0.98	0.75	0.79	0.98	0.75	0.80
DAG	0.99	0.82	0.87	0.99	0.72	0.73	0.98	0.68	0.68
GAT	0.75	0.77	0.82	0.62	0.65	0.69	0.54	0.55	0.61
GCN	0.94	0.82	0.87	0.86	0.75	0.79	0.88	0.75	0.82
LR	0.99	0.81	0.89	0.97	0.76	0.82	0.97	0.77	0.82
SVM	0.99	0.86	0.90	0.97	0.80	0.82	0.97	0.79	0.82
RF	0.99	0.86	0.90	0.99	0.78	0.81	0.99	0.80	0.81
MTC	0.81	0.77	0.84	0.67	0.71	0.68	0.99	0.99	0.99
IRV-MTC	0.82	0.82	0.85	0.75	0.78	0.76	0.88	0.89	0.90
Robust MTC	0.83	0.80	0.85	0.71	0.73	0.71	0.97	0.96	0.99
XGBoost	0.93	0.84	0.88	0.85	0.76	0.80	0.82	0.73	0.84

### 3.3 Optimization Led to Comparable Performances of all Models

Hyperparameters tuning of the selected models led to the identification of the combination of parameters that maximizes the model’s ROC-AUC score. The detailed optimization results, the retained configurations for each model, and the corresponding performances in terms of ROC-AUC, accuracy, F1-score, MCC, and Cohen’s Kappa coefficient were reported in Supplementary Table S3. Learning rates, dropout, and the number of learned features appeared to be the most influential parameters on model performances. In fact, the optimal thresholds for the GCN model were a learning rate of 0.001 and a dropout of 0.1. For the DAG model, the optimal learning rate was 0.0005, and the number of learned features per atom in the graph was equal to 30. The optimal batch size and number of epochs for both models were 64 and 40, respectively (Supplementary Table S3).

**FIGURE 3:**
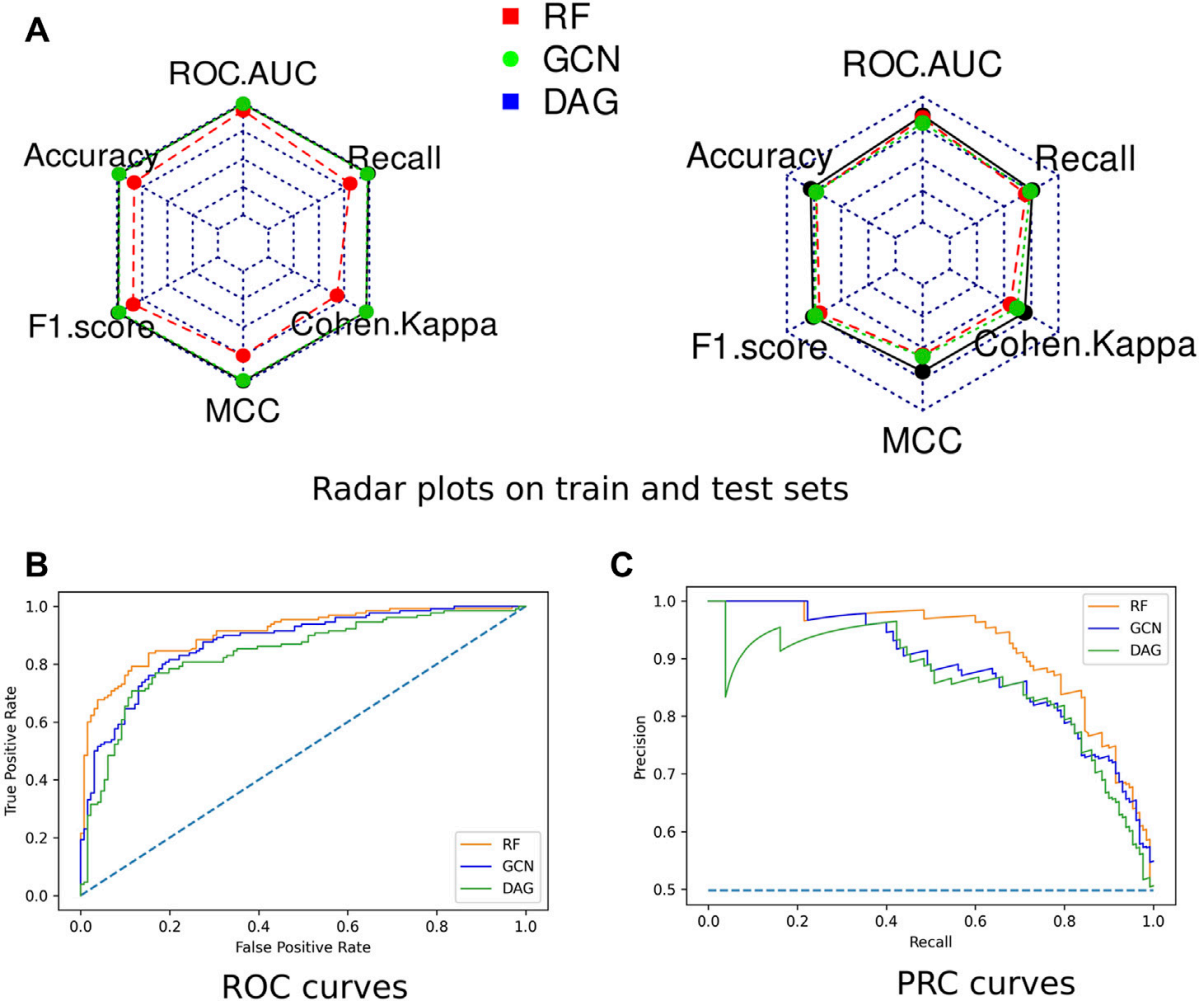
Performances of the optimized models. **(A)** Radar plots of the models’ performances assessed on the train set **(left)** and the test set **(right)** through ROC-AUC, F1-score, Accuracy, Cohen’s Kappa, MCC, and Recall. **(B)** The ROC curve of all three models. **(C)** The Precision-Recall (PR) curve of all three models.

Radar plots representing all computed scores for each model on the train and test sets were generated (Figure 3). None of the algorithms presented an overfitting trend. They all exhibited round-shaped radar plots indicating no differential performance based on the different scoring metrics. Overall, the RF algorithm slightly outperformed both DL algorithms. All three models presented MCC values higher than 0.5, indicating their ability to provide a satisfying class prediction for anticoronavirus molecules (Supplementary Table S3). The RF and DAG models exhibited Cohen’s Kappa coefficient higher than 0.6, which indicates the substantial power of these algorithms in distinguishing both classes. The GCN model presented a coefficient value equal to 0.56, indicating a fair interrater power.

The Receiver Operating Characteristic (ROC) curves exhibited smooth exponential-like shapes for all models, indicating satisfying classification power. The Precision-Recall (PR) curves also presented fair shapes for a balanced dataset (Figure 3). At last, we performed a tenfold cross-validation. The average values of ROC-AUC, the F1-score, and the Recall over ten iterations were reported with the standard deviation values in Table 2. RF kept exhibiting the highest scores, although values were comparable across the three models. GCN achieved a higher ROC-AUC score as compared to the DAG model, an equivalent F1-score, and a lower Recall. Our results so far indicated that DL models kept achieving scores slightly lower than those of RF, despite being comparable.

**TABLE 2:** Tenfold cross-validation results for the best classifiers. Scores are presented as mean values ± SD based on 10 iterations.

Model	ROC-AUC	F1-score	Recall
RF	0.85 ± 0.026	0.78 ± 0.027	0.76 ± 0.032
DAG model	0.79 ± 0.013	0.73 ± 0.052	0.74 ± 0.103
GCN model	0.83 ± 0.026	0.73 ± 0.037	0.70 ± 0.082

### 3.4 GCN Model Demonstrated Noticeable Generalization Power

The last validation step was performed on the three optimized algorithms in order to assess their predictive power in identifying lead compounds against coronaviruses in general and SARS-CoV-2 in particular. Considering the heterogeneity of our dataset in terms of experiments and targets, it is important to assess the ability of the AI algorithms to generalize when tested on unseen datasets. To this end, we split our dataset into category-based subsets. Only categories 3CLpro_Cov and PLpro_Cov presented sufficient data points (Supplementary Table S1) to be used for a stratified validation of the algorithms’ performances.

Homogeneous training denotes all experiments where models were trained and tested on one category subset. Heterogeneous training denotes all experiments where models were trained on the mixed dataset and tested on one category subset. Finally, we called mixed training the experiments where models were trained and tested on the dataset consisting of a mix of categories. Performances in terms of accuracy, F1-score, Recall/sensitivity, and specificity were reported in Supplementary Table S4.

Algorithms’ performances on the 3CLpro_cov category presented comparable values with the mixed training results. On the other hand, low Recall values were obtained with the PLpro_cov category trained on homogeneous and heterogeneous data (Supplementary Figure S1). It is noteworthy to report that the 3CLpro_cov subset constitutes 41.6% of the mixed dataset and presents equivalent proportions between the “active” and “inactive” classes. This was not the case for the PLpro_cov subset, which constitutes 35.8% of the mixed dataset but presented nonequilibrated class distribution (71.0% of inactive molecules). This can explain the low Recall scores obtained for this particular category (Supplementary Figure S1).

**FIGURE 4:**
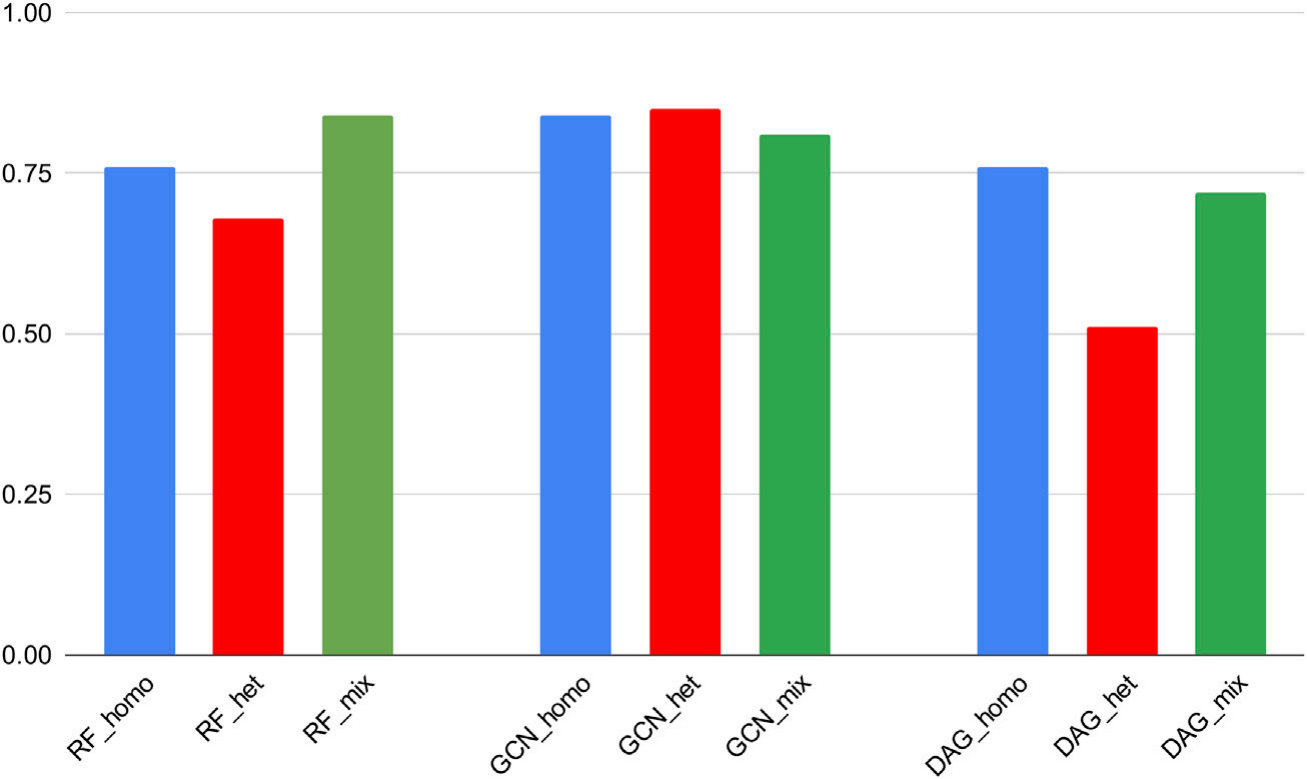
ROC-AUC scores of the best classifiers tested on stratified subsets of the data (homogeneous, heterogeneous, and mixed).

Noticeably, RF and GCN models could achieve comparable Recall scores through the homogeneous and heterogeneous training experiments. This means that these algorithms exhibited a similar ability to correctly predict active molecules if trained either on the mixed dataset or on the subset of the 3CLpro_cov category and then tested on the 3CLpro_cov test set. In addition, the GCN scores were maintained close to those obtained on the mixed dataset and in comparison with cross-validation results (Figure 4). This revealed a generalization power of this particular DL algorithm superior to the other models.

In order to confirm such findings, we performed an external validation of the three algorithms’ ability to predict potential inhibitors targeting SARS-CoV-2 out of the FDA-approved drugs collection. We used a PubChem bioassay that consisted in a primary screen of 1,518 FDA-approved molecules against SARS-CoV-2-infected cells, out of which 17 molecules were retained as potentially active. Out of our mixed dataset, we removed all molecules included within this external validation set. We retrained all three models on our mixed dataset using its full content. Then, we predicted for all FDA-approved molecules from the validation set their activity class. We assessed the classification outcome in comparison with the experimental data and calculated the confusion matrix elements (TP, TN, FP, and FN) for each model under two scenarios (Table 3). First, we calculated the confusion matrix elements while comparing the predicted activity class without regard to the classification confidence (Supplementary Table S5). Then, we applied a threshold of 80% confidence to select the molecules that would be prioritized by each algorithm. Examining this set of prioritized molecules shall assess the usefulness of our classifiers in providing a successful subselection of molecules for experimental validation.

**TABLE 3:** External validation of the three models’ performances in comparison with experimental results from the PubChem bioassay AID_1409594. Columns 2–5 report TP, TN, FP, and FN counts based on the overall predictions of the algorithms. Columns 6–9 report the TP, TN, FP, and FN counts based on the subselection of molecules with prediction confidence higher than 80%.

Activity criterion	All molecules: no confidence threshold				Subselection of molecules above the 80% confidence threshold			
	TP	TN	FP	FN	TP	TN	FP	FN
RF	4	490	119	13	1	425	12	8
DAG	7	719	340	10	3	359	99	3
GCN	8	877	182	9	5	835	147	9

For each algorithm, we first observed the TP and FN counts out of the 17 active molecules. Overall, the GCN model achieved the highest TP count of 8/17 and the lowest FN count of 9/17. The next best performer was the DAG model with TP counts of 7/17, while RF demonstrated the lowest TP count of 4/17 (Table 3). Interestingly, when considering the prioritized list of molecules using the 80% selection threshold, the GCN model achieved the best performances with most of the TP being within the priority list (5 out of 8). The same trend was observed for the TN count with 835/877 being correctly classified as inactive with confidence higher than 80%. Less satisfying rates were achieved by the DAG model (3/7 of TP and 3/10 of FN within the 80% confidence threshold selection) and RF (1/4 of TP and 8/13 of FN within the 80% confidence threshold selection). Thus, the GCN model demonstrated a higher ability to correctly classify both active and inactive molecules within the FDA-approved drugs collection.

## 4 Discussion

AI, precisely ML and DL, have now demonstrated high potential of delivering successful research outcomes in the field of DD (Achdout et al., 2020; Gupta et al., 2021). The application of ML algorithms to cheminformatics and DD is heavily dependent on the rise of molecular encoding systems. The early descriptors consisted in a series of physicochemical properties of the molecules that rapidly demonstrated their limitations. Thus, chemical structure encoding appeared as a promising venue with the underlying hypothesis that the activity of a molecule is heavily correlated with its chemical structure (Gfeller et al., 2014; Shi et al., 2015; Perualila-Tan et al., 2016). Multiple approaches dedicated to calculate molecular fingerprints were then proposed (Bero et al., 2017). These consist in capturing topological and connectivity information within the molecule structure for an enhanced description as compared to simple physicochemical descriptors. Other groups proposed graph convolution-based algorithms that consider the molecule structure as an undirected graph where atoms are nodes and bonds are vertices. These methods were readily useful to implement DL architectures toward DD (Zitnik et al., 2018; Li et al., 2019; Zhang et al., 2019). Conventional ML methods such as RF, SVM, and simple NN demonstrated their ability to predict the inhibitory activity of molecules (Heikamp and Bajorath, 2014; Cano et al., 2017) in the particular case where datasets are limited to a few hundred molecules. On the other hand, DL algorithms achieved interesting results on larger datasets (Unterthiner et al., 2014; Aliper et al., 2016; Lenselink et al., 2017). This reflects the consistent dependency of DL algorithm performances on data size, although they are noticeably gaining ground, exhibiting as high performances as classic ML algorithms (Gupta et al., 2021; Walters and Barzilay, 2021). As DD is a low-data domain, adapted DL approaches were proposed such as one-shot (Altae-Tran et al., 2017) and few-shot (Liu et al., 2021) learning methods based on structure-activity relationships for activity predictions. Compared to more classical approaches, they demonstrated higher predictive power using a small number of positives in their training sets. However, they showed poor capability of generalization to distinct datasets.

In the present work, we assessed the performances of seven ML algorithms and four DL algorithms in predicting the activity of molecules against the COVID-19 viral agent. The training data is a unique collection of 2,610 data points integrated from different sources. It includes molecules presenting inhibiting actions against SARS-CoV and SARS-CoV-2 through multiple and heterogeneous experiments. Our results demonstrated the usefulness of such a dataset in building ML algorithms for activity prediction tasks toward DD against COVID-19. Best performing algorithms, namely, GCN and RF, demonstrated stable performances across different training/testing simulations on stratified subsets of the data. Through external validation on unseen data, the GCN model demonstrated the highest predictive power overall. The MoleculeNet authors performed an extensive benchmarking of multiple ML/DL algorithms, including those studied herein on different tasks and datasets (Wu et al., 2018). RF and the GCN model were tested on multiple datasets (biophysics, physical chemistry, physiology, and quantum mechanics) and were often identified as the best performing algorithms within the conventional methods and the graph-based methods, respectively (Wu et al., 2018). This is in line with our findings, although no direct comparison is possible due to the difference in the datasets used and the tasks on which performances were evaluated.

Data have always been a determinant factor in delivering robust ML. In the field of DD, it is a constant challenge to overcome. Many groups made considerable efforts in constituting dedicated datasets for DD (Gaulton et al., 2012; Yang et al., 2021). The interest in merging data from multiple sources (projects, experiments, etc.) was explored by other groups (Duran-Frigola et al., 2019; Zeng et al., 2019; Irwin et al., 2020). Irwin et al. demonstrated the ability of the Alchemite, a state-of-the-art DL algorithm, to outperform the RF-based QSAR model in property prediction (Irwin et al., 2020). A recent work described a database called D3Similarity that contains 603 molecules with a validated activity against coronaviruses or human receptors (Yang et al., 2021). The database has a web interface that allows for the screening of novel ligands to predict their potential to affect one of the main targets of SARS-CoV-2, namely, the 3CLpro and the PLpro. The activity prediction is performed through a direct assessment of the 2D or 3D similarity of a target molecule to the database elements. In this context, we have deployed important efforts in collecting and curating a dataset that can serve in training and validating different ML and DL approaches in tackling the search for therapeutics against SARS-CoV-2. Our dataset is larger than the D3Similarity dataset and yet ready for use in ML/DL applications against SARS-CoV-2. Conversely, it does not account for quantitative activity information.

It seems important to engage further efforts to integrate more information in our dataset toward its use for a quantitative prediction of molecules activity. Moreover, a deeper analysis of the dataset content may reveal important knowledge for DD projects. Further tuning of the dataset will aim to integrate valuable knowledge on what to expect from effective anti-SARS-CoV-2 molecules (Tummino et al., 2021). In fact, it has been demonstrated that the cationic amphiphilic nature of some drugs may induce phospholipidosis rather than actual antiviral effects (Tummino et al., 2021). Such properties should be further examined to enhance the relevance of our dataset to the development of COVID-19 therapeutics.

## 5 Conclusion

In the present study, we collected and curated a dedicated dataset of 2,610 molecules having anticoronavirus effects. This valuable resource was formatted and used to perform different simulations and optimization of eleven ML and DL algorithms toward the classification of molecules into active and inactive classes. We were able to obtain three highly accurate classifiers that were validated through cross-validation and on an external set of data. The DL algorithms demonstrated the best performances.

## Data Availability

The datasets presented in this study along with the jupyter notebooks can be found online on https://github.com/Harigua/ML_DD-applications/tree/main/COVID-19.

## Supplementary Material

Supplementary Figure S1 Performances of the three algorithms GCN, DAG and RF assessed on homogeneous vs. heterogeneous data. (a) Performances for the subsets of the category 3CLpro. (b) Performances for the subset of the category PLpro. (c) Comparison between the models performances on the mixed dataset and the 3CLpro subset.

Supplementary Table S1 Statistics on the anticoronavirus dataset according to their origin (bioassays vs. literature) and their type (experiments, targets, etc). Composition of the training, validation and test set in terms of active and inactive molecules are indicated.

Supplementary Table S2 Performances of all 11 algorithms using different splitting ratios (80/10/10 vs. 60/202/20) and methods (random vs. scaffold split) on different datasets (heterogenoeous vs. homogeneous).

Supplementary Table S3 Hyperparameters’ tuning and optimization for the three best performers: GCN, DAG and RF.

Supplementary Table S4 Performances of the optimized algorithms GCN, DAG and RF in terms of accuracy, F1-score, sensitivity and specificity, on different subsets of the dataset.

Supplementary Table S5 Predictions assessment of the three algorithms GCN, DAG and RF on the subset of experimentally validated molecules (sheet 1). Sheets 2-4 contain the prediction outcomes for each algorithm for all molecules.
