# Supplementary figures and images for: Deep Learning Algorithms Achieved Satisfactory Predictions When Trained on a Novel Collection of Anticoronavirus Molecules
Source: Front Genet. 2021 Nov 29;12:744170. doi: 10.3389/fgene.2021.744170 (PMC8667578; doi:10.3389/fgene.2021.744170)

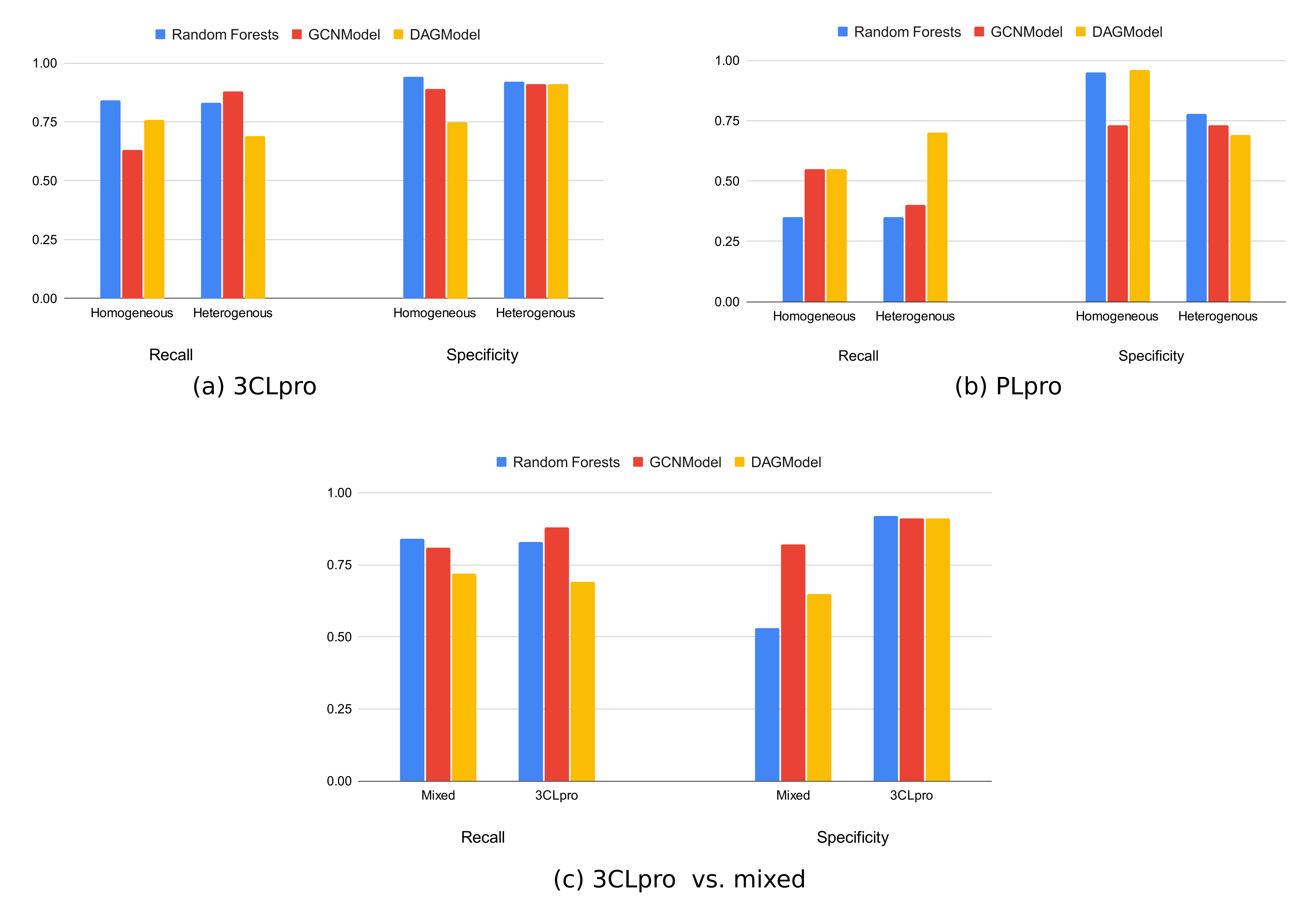

Supplement: Supplementary file 5 [file Image1.PNG]
